# Are we happy with our work in globalization? Globalization experience, achievement motivation, and job seniority as predictors of work satisfaction in a group of office workers

**DOI:** 10.1186/s12992-023-00941-w

**Published:** 2023-06-21

**Authors:** Jakub Filipkowski, Romuald Derbis

**Affiliations:** Institute of Psychology, Opole University, Opole, Poland

**Keywords:** Globalization experience, Goal achievement, Work satisfaction, Job seniority

## Abstract

**Background:**

The main aim of this study was to determine whether globalization experience is a predictor of work satisfaction. In addition, we inspected a regression model consisting of globalization experience, job seniority, and goal achievement to determine how much variance in work satisfaction is accounted for by globalization experience. Most the theoretical texts about globalization suggest its negative impact on everyday life. The negative effects are – work–life balance problem, weakening of mechanisms to protect against the fear of death, and uncertainty.

**Method:**

250 office workers participated in the study (*M*_*age*_ = 38.37; 145 females and 105 males). They responded to paper-and-pencil anonymous questionnaires measuring globalization experience, achievement goals, and work satisfaction. Respondents were also asked about their job seniority. We used Spearman’s rho correlations and multiple linear regression to check the basic linear relation between variables, and hierarchical multiple regression to determine which of them is the strongest predictor of work satisfaction.

**Results:**

The results indicated that globalization experience (R^2^ change = 0.089; p < .05) is a statistically significant negative predictor of work satisfaction and job seniority (R^2^ change = 0.056; p < .05) while achievement goals (R^2^ change = 0.188; p < .001) are positive predictors of work satisfaction.

**Conclusion:**

We concluded that further research on globalization experience is necessary because it is the precursory individualistic approach to globalization research and we obtained a statistically significant yet small relation with work satisfaction in correlation and regression analyses. The presented results are also the rationale for promoting mastery approach goals in the workplace to improve work satisfaction as they are statistically significant positive predictors of it.

## Key messages

Globalization experience is negatively related to work satisfaction. Especially the impact of globalization on the sense of security may affect the workers’ contentment.

Achievement motivation is positively related to work satisfaction. Motives related to striving for mastery are the most important predictor of workers’ contentment.

Time spent in a certain company is positively correlated with work satisfaction.

## Introduction

Globalization may be described as a set of processes leading to the creation of a global cosmopolitan society [1]. There are numerous sociological and philosophical deliberations about the impact of globalization on our lives and work [1–5], yet relatively few empirical studies have considered this topic to date from a psychological individualistic perspective [6–9]. Many of them suggest a negative impact, for example, globalization is correlated with exhaustion in the work environment [10] and may be the cause of increased stress or a permanent need to be on standby [11]. There is, however, still a problem with the psychological approach to globalization because of the lack of tools to measure how people experience it. Some methods that have been used include questions about friends abroad and travels [7], trade policy and flow [11], and watching international TV channels [12]. In this study, we used the Globalization Experience Model (GME) [13], which describes affective and cognitive experiences of globalization processes on self-realization, security, and sense of agency, to test whether individual exposure to globalization predicts work satisfaction. Apart from negative globalization experience, we examined two variables that may be important in understanding its relation with work satisfaction – achievement goals [13] and job seniority.

Probably the first scholar who tried to conceptualize this term in the context of psychology was Sampson [14]. He claimed that we turned from a modern to a postmodern society. Globalization is connected with the weakening relevance of traditional values and the diminishing importance of small communities which used to give us safety. This approach is similar to the Folk Theory of Social Change [9]. According to this theory, globalization changes the values people deem important, away from those that are geared toward family and building relations, to those more related to competencies. Moreover, Kashima et al. [9] imply that processes connected to globalization are a permanent feature of human civilization and every person must adapt to them. Since these processes are inexorable, people like to reminisce about past times regardless of the era in which they live. Globalization changes might be dangerous if we accept Terror Management Theory [15]. It states that we are able to live with the awareness of imminent death because of traditional culture and rituals. They help us to make our lives meaningful and not limited to its physical sphere. Salzman [16] suggested that globalization may lead to cultural trauma because it devaluates the meaning of traditions. As we stated earlier, there are not many operationalizations of globalization in psychology. One of them is Derbis, Pajestka, and Jasiński’s [6] GME. Authors have adopted an individualistic approach and focused on personal experiences of the cognitive and affective impact of universalization, identity problems, compulsion to develop, and other factors, in their everyday life. In contrast to most of the theories, GME emphasizes the negative impact of globalization on individuals, not the environmental context and global processes. Authors of GME insinuate that this experience is mostly harmful, implying that the stronger the globalization experience, the more detrimental to an individual its impact on self-realization, life, and sense of security [6].

Research on work satisfaction began with studies that focused on its connection to efficacy at work. Researchers concluded that there is a positive relation between positive feelings at work and efficacy [17]. They noticed that material aspects of employment are not enough to satisfy workers [17, 18]. Most definitions of work satisfaction focus mostly on positive affects [19–21], but we believe that the cognitive evaluation of professional life is a more suitable way to assess the relations with globalization. We are aware that cognition is strongly affected by emotions [21] so cognitive evaluation of professional life is also affected by emotional attitudes toward it. Zalewska [22] approached the measure of work satisfaction in a way similar to global satisfaction with life [23], by using cognitive global assessment, in which people choose their own criteria to rate their previous achievements. We can consider different aspects in evaluating our work, and they may be interesting in specific research contexts, but global assessment is more sensitive in studies that endeavour to find its determinants or identify outcome variables, as Zalewska suggests [22]. In times of globalization, the average working time in the United Kingdom has decreased and stabilized at 40 h per week but, despite this, there is an increasing percentage of people who work more than 48 h per week [2]. More recent data provided by the World Health Organization show that people usually work 30 to 34 h per week, but in 2016 8.9% of people were exposed to long working hours, more than 55 h per week [24]. In Poland, the average time of work is also stabilized at 40 h per week [25]. The problem of combining work and private life became so common that it is now identified as *work-life balance* and there are popular practices related to it [26]. It is now less important to be engaged in work than it used to be, but we have to be ready to learn new things or even change occupations, we have no guaranteed job security [27]. On the basis of the above, the exposition to long working hours, the *work-life balance problem*, and the decreased job security, we anticipated a potential detrimental relationship between work satisfaction and a negative globalization experience.

We believe it is possible that work satisfaction is correlated with other variables, inter alia, motivational and seniority related. These characteristics in turn may be related to a negative globalization experience. This means they might explain the same part of work satisfaction variance, thus adding these variables may enhance the understanding of the mechanisms underlying the relationship between negative globalization experience and work satisfaction. Consequently, we added examples of such constructs to our theoretical and empirical models. To assess the impact of motivation, we proposed goal achievement motivation [28] and years of occupational seniority, along with years spent in the current job, in order to evaluate the relationship between seniority and work satisfaction. According to Elliot and McGregor [29], there are four achievement goals representing two bipolar dimensions: competence and valence. A *mastery-approach* goal involves concentration on competence and searching for the best problem-solving strategies [30]; *mastery-avoidance* involves an evasion of building skills to help one meet current challenges, or losing them [29]; *performance-approach* is a focus on performance that may be noticed by others, and *performance-avoidance* involves withdrawing from tasks that may be too challenging or out of a fear that the result would be evaluated negatively [30]. A mastery-approach goal is considered the most efficacious and healthy, but performance-centered ambitions may also be adaptive [31]. There is also a wealth of research that has shown a positive relation between work satisfaction with engagement [32], general work motivation [33], and achievement orientations [34]. Moreover, there is a suggestion, that the impact of globalization, may strengthen competitive and mastery goals [35]. An analysis of the literature indicated that time spent at one company may be a positive correlate of work satisfaction [36]. However, the relationship may not be linear but *U* shaped. Job tenure is a more consistent predictor of work than age [37], which is generally considered a positive predictor of work satisfaction [36–38]. Some authors underline the difference between measures of job seniority; there may be distinct effects for time spent as a worker in one occupation and years of devotion to one employer [39]. Hereafter, we use the term “occupational seniority” to describe the overall time one has worked in a certain occupation, and “job seniority” to refer to years of employment at one’s current place. We found these variables important also in accordance with globalization, as it is possible, that workers with short tenure, are most likely to experience its negative effects [39].

According to the abovementioned points, our hypotheses are:

### H1

Negative globalization experience is a negative correlate of work satisfaction.

### H2

A model consisting of negative globalization experiences, goal achievement motivation, and job seniority predicts work satisfaction.

### H2.1

Negative globalization experience is a negative predictor of work satisfaction.

### H2.2

Job seniority is a positive predictor of work satisfaction.

### H2.3

Goal achievement motivation is a positive predictor of work satisfaction.

## Materials and methods

### Procedure

We used a cross-sectional design. Participants were given questionnaires and asked to fill them with correct information. They were also asked about their consent to take a part in a research project about job seniority, occupational seniority, and age. The survey was conducted in the pencil-and-paper method.

### Sample

Research participants were professionally active Polish workers who did most of their work in office space. We chose this group because, in such an occupation, workers are more exposed to manifestations of globalization than other professions, e.g., employees from other countries, part of production is held outside the organization, and people have to communicate in their non-native language [40]. There were 250 participants (145 females and 105 males). Their average age was 38.37 years (ranging from 22 to 61), and the average seniority at their current workplace was 7.83 years. Most of them described themselves as managers (15.6%), inspectors (12%), or specialists (12%). According to the a priori sample size calculator for multiple regression^1^, the minimum sample to detect the medium size effect in our model is 118 [41]. We used nonprobability sampling with being an office worker as the only criterion. The survey was conducted with the help of external services specialized in this kind of research.

### Measures

To measure the negative impact of globalization on one’s life, we used the Globalization Experience Scale (GES) [6]. It defines globalization experience as emotional and cognitive reactions to globalization processes, not the exposure to them or attitudes towards the concept of globalization. GES measures three aspects of negative globalization experience: (1) self-realization, (2) influence on life, and (3) sense of security. Participants were asked to rate their agreement with 14 statements on a scale that ranged from 1 (*it happens to me very occasionally/never*) to 5 (*to a very large extent*). The GES is an internally consistent measure in original validation (Cronbach’s α = 0.80 for the summary score and 0.69–0.79 for subscales). Reliability measure is satisfactory also in the current study for summary score (Cronbach’s α = 96; CI = 0.95; 0.97) as well as for subscales – sense of security (Cronbach’s α = 0.94; CI = 0.92; 0.95), influence (Cronbach’s α = 0.92; CI = 0.90; 0.94) and self-realization (Cronbach’s α = 0.90; CI = 0.88; 0.92).

Work satisfaction was measured with the Work Satisfaction Scale [22], which is directly modeled on the Satisfaction with Life Scale [23]. It is defined as a global cognitive assessment of one’s work life. Participants rate their responses to five questions on a scale that ranges from 1 (*strongly disagree*) to 7 (*strongly agree*). In the original validation study, the WSS turned out to be reliable (Cronbach’s α > 0.80). It was also reliable in the current study (Cronbach’s α = 0.94; CI = 0.92; 0.95).

To measure achievement goals, we used the Polish translation [42] of the Achievement Goals Questionnaire–Revised (AGQ–R) [43]. It measures four approaches to achieving success: mastery-approach, mastery avoidance, performance approach, and performance-avoidance. Participants were asked to rate their agreement with 12 statements on a scale that ranged from 1 (*strongly disagree* to 7 (*strongly agree*). In yhe Polish version of the AGQ–R internal consistency is not satisfactory in some scales (Cronbach’s α = 0.49–0.88) but it is reliable in the current study - mastery-approach (Cronbach’s α = 0.89; CI = 0.86; 0.91), mastery avoidance (Cronbach’s α = 0.89; CI = 0.86; 0.91), performance approach (Cronbach’s α = 0.82; CI = 0.78; 0.85), and performance-avoidance (Cronbach’s α = 0.88; CI = 0.86; 0.91).

To measure work seniority, we asked participants how long they had been professionally active and how long they had worked for their current company. They provided exact numbers of years.

Reliability coefficients in the current study were calculated with SPSS 28 (Statistical Package for Social Sciences). We took the value of 0.70 of Cronbach’s α as satisfactory [44].

### Statistical analyses

To analyze the data, we used SPSS 28 and jamovi software version 2.3 [45]. Before calculating statistical analyses related to hypotheses, we computed descriptive statistics with normality tests, skewness, and kurtosis [46] for all variables to determine whether the distribution is normal, non-parametric Spearman’s rho correlation, and checked the assumptions for regression analyses – variance inflation factor [47] and a Durbin-Watson test [48] for autocorrelation. Next, we performed multiple regression to check if negative globalization experience is a statistically significant predictor of work satisfaction considering also two-way interactive effects. Furthermore, we calculated hierarchical regression analysis, which allows to enter the set of variables in each step, to determine more nuanced mechanisms underlying the relation of negative globalization experience with work satisfaction and compare the percentage of explained variance to age-related variables and achievement goals. We assumed a 0.05 alpha level but according to its problematic nature [49] we reported exact p-values in the most important results and effect size coefficients for regression analyses [50].

## Results

We calculated basic descriptive statistics and checked the assumption of distribution normality by the Shapiro-Wilk test (Table 1).

**Table 1:** Descriptive statistics of job seniority, work satisfaction, negative globalization experience, and goal achievement motivation with the Shapiro-Wilk normality test

Variable	*Mean*	*Mdn*	*SD*	Min	Max	Skewness	Kurtosis	*W*	*Shapiro-Wilk’s p*
Occupational seniority	12.93	12.00	8.67	1.00	39.00	0.571	–0.483	.943	< .001
Job seniority	7.83	7.00	5.50	0.50	27.00	0.910	0.507	.923	< .001
Work satisfaction	19.78	20.00	6.49	5.00	33.00	–0.204	–0.816	.975	< .001
Negative globalization experience	18.35	14.00	8.49	13.00	66.00	2.369	6.048	.597	< .001
Sense of security	7.67	6.00	3.59	5.00	30.00	2.616	8.023	.554	< .001
Influence	5.20	4.00	2.54	4.00	17.00	2.339	4.950	.547	< .001
Self-realization	5.48	4.00	3.02	4.00	19.00	2.006	3.172	.560	< .001
Mastery approach	12.82	13.00	2.20	3.00	15.00	–1.151	1.580	.864	< .001
Performance approach	11.99	12.00	2.77	3.00	15.00	–1.251	1.440	.868	< .001
Mastery avoidance	12.85	13.00	2.27	3.00	15.00	–1.272	1.806	.850	< .001
Performance avoidance	12.20	12.50	2.70	3.00	15.00	–1.326	1.828	.859	< .001

All variables proved to be not normally distributed. In addition, for negative globalization experiences and associated subscales, skewness was bigger than 2, which means that the distribution is asymmetric so non-parametric correlation should be used. Accordingly, in the next step we decided to use Spearman’s rho (Table 2).

**Table 2:** Spearman’s rho correlation coefficients for job seniority, work satisfaction, negative globalization experience, and goal achievement motivation

	1	2	3	4	5	6	7	8	9	10	11
1	—										
2	.129*	—									
3	.158*	.950***	—								
4	.215***	.679***	.652***	—							
5	–.132*	.099	.108	.136*	—						
6	–.155*	.125*	.132*	.127*	.908***	—					
7	–.145*	.162*	.172**	.159*	.883***	.899***	—				
8	–.113	.012	.030	.024	.862***	.725***	.671***	—			
9	.508***	–.003	.005	.118	–.208***	–.236***	–.259***	–.159*	—		
10	.059	.041	.042	–.022	–.148*	–.146*	–.153*	–.118	.294***	—	
11	.378***	.034	.015	.107	–.169**	–.222***	–.207***	–.121	.802***	.280***	—
12	–.049	–.000	.014	–.016	–.110	–.120	–.125*	–.075	.167**	.749***	.219***

*Note*. *p < .05. **p < .01. ***p < .001.

1 – Work satisfaction; 2 – Age; 3 – Occupational seniority; 4 – Job seniority; 5 – Negative globalization experience; 6 – Sense of security; 7 – Influence; 8 – Self-realization; 9 – Mastery approach; 10 – Performance approach; 11 – Mastery avoidance;

12 – Performance avoidance.

It turned out that work satisfaction was correlated positively with age, job seniority, occupational seniority, mastery approach, and mastery avoidance, and negatively with negative globalization experience along with the associated subscales: sense of security, self-realization, and influence. Mastery approach and mastery avoidance were the strongest correlates.

The next step to test our hypotheses was a multiple linear regression analysis to assess whether negative globalization experience is a predictor of work satisfaction and a hierarchical multiple linear regression to determine whether adding negative globalization experience to the model predicting work satisfaction with work seniority and achievement goals improved it in a statistically significant way. We checked the assumptions: multicollinearity by a variance inflation factor (Table 3) and autocorrelation by a Durbin–Watson test. It appeared that there is a statistically significant but not strong autocorrelation between examined variables (Durbin–Watson = 1.70, autocorrelation = 0.151; *p* = .010) and the variance inflation factor did not exceed a value of 10, which is acceptable.

**Table 3:** Variance inflation factor (VIF) for a regression model with work satisfaction as a dependent variable

Variable	VIF	Tolerance
Job seniority	1.81	.554
Occupational seniority	8.00	.125
Age	8.52	.117
Sense of security	8.93	.112
Influence	7.48	.134
Self-realization	2.36	.423
Mastery approach	3.31	.302
Performance approach	3.97	.252
Mastery avoidance	3.17	.315
Performance avoidance	3.87	.258

The next step to determine the predictive role of a negative globalization experience in relation to work satisfaction was preparing a linear regression model with the GES subscales sense of security, influence, and self-realization as independent values and work satisfaction as the dependent variable, considering two-way interactions as well (Table 4).

**Table 4:** Standarized beta coefficients for multiple linear regression with two-way interactions of work satisfaction with negative globalization experience as a predictor

Predictor	
Sense of security	–.273*
Influence	.012
Self-realization	.011
Sense of security × Influence	.162
Sense of security × Self-realization	.177
Influence × Self-realization	–.347*
*F*	4.86*
*R* ^2^	.056
Cohen’s *f*^2^	.059

*Note*. *p < .05. **p < .01. ***p < .001.

The model predicted the level of work satisfaction in a statistically significant way; it covered 5% of the variance. The effect size was small (Cohen’s *f*^2^ = 0.05). The most important predictors were a sense of security (*Β* = –0.273; p = .025) and the interaction of influence and self-realization (*Β* = –0.347; p = .014), both negatively correlated with work satisfaction.

Afterward, we calculated a hierarchical multiple regression with age, occupational seniority, and job seniority in the first step, on the grounds that they are not psychological variables; negative globalization experience in the second step; and goal achievement in the last one, because they were the strongest correlates of work satisfaction (Table 5).

**Table 5:** Standarized beta coefficients for hierarchical linear regression of work satisfaction as the dependent variable with seniority traits, GES scales, and achievement goals as predictors

	Step 1	Step 2	Step 3
Job seniority^a^	.230**	.237**	.151*
Occupational seniority^a^	.339	.379*	.364*
Age	–.374*	–.388*	–.330*
Sense of security		.011	–.062
Influence		–.220	–.044
Self-realization		.040	–.005
Mastery approach			.474***
Performance approach			.013
Mastery avoidance			–.051
Performance avoidance			–.149
*F*	4.86**	3.97***	9.17***
*R* ^2^	.056	.089	.277
Cohen’s *f*^2^	.059	.097	.383
*R*^2^ change		.033*	.188***

*Note*. *p < .05. **p < .01. ***p < .001.

^a^In years.

Analyses showed that adding every set of variables statistically significantly changed the prediction of work satisfaction: Step 1 with age, job seniority, and occupational seniority, *F*(3, 246) = 4.86, *p* = .003, *R*^2^ = 0.056; Step 2 with the added three dimensions of negative globalization experience (sense of security, influence, and self-realization), *F*(6, 243) = 3.97, *p* < .001, *R*^2^ = 0.089, *R*^2^ change = 0.033; Step 3 extended with four achievement goals (mastery approach, performance approach, mastery avoidance, and performance avoidance), *F*(6, 243) = 9.17, *p* < .001, *R*^2^ = 0.277, *R*^2^ change = 0.188. Negative globalization experience was a statistically significant predictor that accounted for 10% of the variance, and the strongest predictor was goal achievement motivation, which predicted 19% of the variance. The strongest single predictor was mastery approach (β = 0.474; *t* = 4.37; *p* < .001). Together, all of the proposed variables accounted for 28% of work satisfaction variance.

## Discussion

Job seniority and professional activities were positive correlates and, along with age, statistically significant predictors of work satisfaction. The results related to time spent at one company or in one occupation are consistent with previous empirical reports [37, 38]. We speculate that new workers feel more pressured by a workplace environment that is new to them. People who just started a career need to learn new skills, inter alia by mistakes, whereas more experienced workers can focus on displaying expertise. They also may feel it will be easier to find a new job when they feel competent [51]. Overall, occupational seniority was more strongly correlated with work satisfaction than job seniority. This may be connected to wages; contrary to common beliefs, job seniority in one company is not strongly correlated with earnings [52]. Most of the changes in payment are due to re-employment. Interestingly, in a model that considered job seniority and occupational seniority, age was negatively correlated with job satisfaction After including the variance of these two variables, other effects connected with aging (e.g., cognitive problems [53] worsened health, and lower happiness [54] may be negative in predicting work satisfaction. There is also a probability that this result is related to the Polish cultural context; in 1989, there was a transformation of the system that was difficult for some people [55], and a number of them may have never adapted to the new conditions.

Negative globalization experience was correlated negatively with work satisfaction. Its dimensions were also a statistically significant predictor of explained variables, but the effect was small. The most important correlate was sense of security, but multiple regression analyses showed that there was also a statistically significant interaction between a sense of security and influence. It may be that, in times of change, what is most important to our satisfaction is safety: To enjoy our job, we have to be sure that we won’t have to change it the next day. This result may be also explained in light of Herzberg’s Motivation Two-Factor theory - safety is a hygiene factor, which should protect workers from dissatisfaction [18]. The small effect may be caused by an omitted moderator (e.g., job seniority or occupational seniority)^2^ given that the above-mentioned results suggest that a sense of security is an important factor related to work satisfaction. More experienced workers may feel safer about their job. On the other hand, older workers may perceive more changes connected with globalization in their environment because of the broader time perspective. These results are consistent with our predictions and previous theoretical considerations [5, 10, 27]. In addition, we found that negative globalization experience correlates negatively with achievement goals – mastery approach, performance approach, and mastery avoidance, but positively with job seniority. Combining job and occupational seniority in one model with negative globalization experience makes one of its dimensions – sense of security, statistically non-significant. It probably implies the mediation or moderation effect^3^. The relation between job security and work satisfaction may be partially explained or differentiated by the time spent in the company or occupation. This potential effect can be interpreted according to the previous paragraph, by the pressure of being a young worker [51], or problems with adaptation [55]. The small percentage of explained variance may suggest some omitted moderator – maybe the relationship between negative globalization experience and work satisfaction is triggered by the level of some different variable, e.g., self-efficacy [56] or locus of control [57].

Achievement goals turned out to be the most important predictor proposed in this study. The correlation analysis showed that goals connected with competence seeking, mastery approach, and mastery avoidance, were the strongest positive correlates of working satisfaction. The more we focus on our own development, look for new problem-solving solutions, and compare ourselves with a past self rather than other people, the higher our work satisfaction. In the regression model, the most important predictor of all proposed variables was the mastery approach. It is consistent with the achievement goal theory. Its authors suggested initially that the mastery approach is the most adaptive one [30]. Some studies have supported this relation [58]. Adding achievement goals to the model that consisted of GES and seniority variables, does not change the statistical significance of any relation. We can, however, conclude, that achievement goals are a much stronger predictor of work satisfaction than seniority and negative globalization experience. The predominant role of achievement motivation in explaining work satisfaction in comparison to job seniority and globalization experience may be clarified by Maslow’s Theory of Human Motivation. The need for self-actualization is similar to the mastery motive, and it is the highest order, which means that to fulfill it, all the other needs must already be satisfied [59]. A person who is highly motivated to achieve mastery, according to Maslow, should be already happy with what they want. The next explanation of the mastery approach and work satisfaction relationship is Alderferer’s ERG theory (Existence, Relatedness, and Growth) [60] which places growth needs at the peak of Maslow’s motivational hierarchy. Growth leads to satisfaction through the use of personal skills on the way to becoming fully what one can be.

While the research does not directly address health systems, the results may signal some areas worth paying attention to. Antonovsky [61] claimed that most psychological studies were focused on pathology and the dichotomy between “sick” and “healthy” people. In his salutogenic model, he proposed to measure mental health on a continuous scale, suggested that it cannot be treated as a lack of disease, and emphasized the role of this kind of approach to public health. It is important for the subject of our study because job satisfaction is considered one of the markers of mental health in the workplace in the salutogenic model [62]. Thus, the results of our study indicate that suggested predictors – negative globalization experience, achievement motivation, and job seniority, may have an impact on mental health. It is impossible to avoid exposure to globalization processes on individuals, but it may be beneficial to provide further research that will examine possible methods to prepare workers for contact with other cultures and languages and to diagnose whether it is a problem on an organizational level. Studies have shown that goal motives can change during a lifetime, even if there is partial stability in their level [63]. In consequence, there is a possibility that promoting attitudes associated with mastery motives will be beneficial for workers’ mental health. While some studies indicate that changing jobs frequently results in higher pay increases, the results of our research indicate that it is better for work satisfaction to stay in one place for a longer period of time, so it may be worth educating about the impact of this career approach on mental health [64].

There are some limitations to this study that compel caution in interpreting and extrapolating the results. The first one is the weak yet statistically significant autocorrelation of predictors, which can affect estimated coefficients. The next one is a correlational character of analysis with a cross-sectional study design, which reduces the reliability of the results. There is also a probability that some of the effects were caused by the specific Polish cultural context, which lowers the external validity of aur study. So far, the GES has not been translated into English, which makes it impossible to compare our results with other cultures and to determine whether the effects detected in this study also occur in different countries. 19% of explained variance suggests that there are other variables that predict work satisfaction, and they may also alternate the relations from the current study – as mediators or moderators. Based on the presented results, it is impossible to determine how the suggested programs and training aiming to educate about mental health in the context of globalization experience, achievement motives, and career decisions should look. The group of respondents was limited only to office workers, so we are unable to determine whether the potential effects of influencing the experience of globalization, achievement motives, and career decisions, will also take place in groups other than office workers. In future research, the presented model should be extended by behavioral indexes and experimental design to confirm its correctness. To extend the external validity of the result, it may be beneficial to examine the same set of variables in other occupational groups.

## Conclusions

Our hypothesis turned out to be true, and this study provides some information about the nature of job seniority, globalization experience, and goal achievements as predictors of work satisfaction. Job seniority and achievement motivation turned out to be positive predictors of work satisfaction and globalization experience was a negative predictor. The model consisting of all suggested variables explained approximately 19% of the work satisfaction variance. Most of the results are consistent with previous research and theory. The mastery approach appears to be the most important motive from all achievement goals in predicting work satisfaction. Occupational seniority may be a better predictor of work satisfaction than job seniority. The main theoretical impact of our study is connected to the globalization experience. Results suggest that negative globalization experience (especially the sense of security aspect) is a statistically significant predictor of work satisfaction [6]. Based on this, we can conclude that further research on globalization models that reflect mainly on individual experience is reasonable and the assumption about the general negative impact of globalization processes on people’s psyches [6] may be true. On the other hand, the small effect sizes and low correlation coefficients related to GES results suggest the need to rethink the model and revise the measure.

Our results may lead to some practical implications. It is probably important to promote attitudes connected to competence and mastery, more than to approaches targeted at rivalry and results delivery. This may result, seemingly paradoxically, in better efficiency and employee productivity [65]. As it appears, promoting staying in one job for some time may be good for work satisfaction. Globalization experience can be considered a new, hitherto overlooked, problem, that affects our happiness in the workplace. It may be beneficial for workers if greater exposure to its manifestations in some companies would become an interest of practitioners and management.

## Data Availability

The datasets used and/or analyzed during the current study are available from the corresponding author on reasonable request.

## Abbreviations

AGQ–R: Achievement Goals Questionnaire–Revised
ERG theory: Existence, Relatedness and Growth theory
GES: Globalization Experience Scale
SPSS: Statistical Package for Social Sciences
VIF: Variance inflation factor
M: Mean

## Footnotes

1 It is based on desired probability level, number of predictors in the model, anticipated effect size and convenient statistical power level.

2 We performed a post hoc analysis, which showed that the interaction between GES summary score and job seniority was a statistically significant predictor of work satisfaction.

3 We performed a post hoc analysis, which showed that there is no indirect effect of job seniority, occupation seniority, and age on sense of security and work satisfaction relationship. There is, however, a statistically significant moderation – sense of security interacts with job seniority in predicting work satisfaction. Sense of security effect on work satisfaction is statistically significant and detrimental only for average and high levels of job seniority.
